# Bone transport with a unilateral external fixator for femoral infected nonunion after intramedullary nailing fixation: A case control study

**DOI:** 10.1097/MD.0000000000015612

**Published:** 2019-05-17

**Authors:** Chunfeng Liu, Xianghong Zhang, Xiangsheng Zhang, Zhihong Li, Yaozeng Xu, Tang Liu

**Affiliations:** a Department of Orthopedics, The First Affiliated Hospital of Soochow University, Suzhou; b Department of Orthopedics, Suzhou Kowloon Hospital Affiliated to School of Medicine, Shanghai Jiao Tong University, Suzhou; c Department of Orthopedics, The Second Xiangya Hospital, Central South University, Changsha, Hunan; d Department of Orthopedics, Liuzhou General Hospital, Guangxi University of Science and Technology, Liuzhou, Guangxi; China.

**Keywords:** bone transport, debridement and irrigation, external fixator, femoral infected nonunion, intramedullary nailing

## Abstract

This is a therapeutic study to evaluate the results of femoral infected nonunion using bone transport with an external fixator after debridement and irrigation. We retrospectively reviewed 15 patients with femoral infected nonunion after intramedullary nailing fixation of fractures from October 1999 to January 2010 in our institute. There were 7 males and 8 females with an average age of 32.5 years. First, the infection was eradicated completely, and the medullary canals were continuous irrigated for 2-3 weeks. After eradicating the infection tissues, the mean amount of bone defect was 8.7 cm (range, 4.0–16.0 cm). The unilateral consecutive distraction-compression osteosynthesis technique was applied after long-time medullary cavity-wound exclusion surgery. Enumeration data was described by frequency and measurement data by mean. Bone infections were controlled in all patients except 1 patient after the first debridement and irrigation. All patients have achieved bony union without recurrence of infection during the follow-up period, the mean external fixation index was 43.4 day/cm. According to the criteria recommended by Paley, the bone results were graded as excellent in 13 (86.7%) cases and good in 2 (13.3%) cases; the functional results were graded as excellent in 6 (40.0%) cases, good in 6 (40.0%) cases and fair in 3 (20.0%) cases. In management of femoral infectious nonunion which caused by intramedullary nailing fixation, the surgery of consecutive compression-distraction osteogenesis with unilateral external fixator achieves a highly effective treatment, and the method of debridement and irrigation is a compatible choice on the phase of infection-elimination.

## 1 Introduction

Intramedullary (IM) nailing has become a standard procedure for the treatment of both close and open long bone fractures.^[1–4]^ The overall rate of bone infection after IM nailing fixation of long bone fractures was 0.9% to 3.8%^[5,6]^ and the rate of bone infection after high energy open fractures following IM nailing fixation was comparatively higher.^[7]^ Infected nonunion is an infrequent but one of the most challenging orthopedic complications to manage.^[8,9]^ The purpose of this study was to evaluate the safety and efficacy of bone transport with an external fixator after debridement and irrigation in the treatment of femoral infected nonunion after treatment of fractures of femur by IM nailing fixation.

## 2 Materials and methods

### 2.1 Patients

Fifteen consecutive patients were treated in our institute between October 1999 and January 2010 for femoral infected nonunion after IM nailing fixation. There were 7 males and 8 females with an average age of 32.5 years (range, 18–58 years). The injury mechanisms of these patients included 5 falls, 8 traffic accidents, and 2 crush injuries. Three of them, the fracture sites were in proximal femur, 9 in the shaft and 3 in the distal femur. All patients were sustained closed fractures, but the details and classification of the original fractures were not known (Table 1). All the patients received IM nailing fixation as their initial treatment. Among them, 13 nails were originally inserted antegrade and 2 were placed retrograde. The patients had an average of 3.93 surgical procedures (range, 3–6 procedures) before being presented to our hospital. The mean time from the first surgery to admit to our hospital was 25.8 months (range, 14–38 months). When patients were admitted to our institution, 9 cases presented with purulent wound drainage, pain, and/or other signs of infection including fever, wound erythema, and/or elevated markers for infection (white blood cells (WBC), C-reactive protein (CRP), and erythrocyte sedimentation rate (ESR)). Two of the patients had knee joint problem due to their previous walking inability. According to the Judet's^[10]^ classification of nonunion, they were all atrophic nonunions.

**Table 1:**
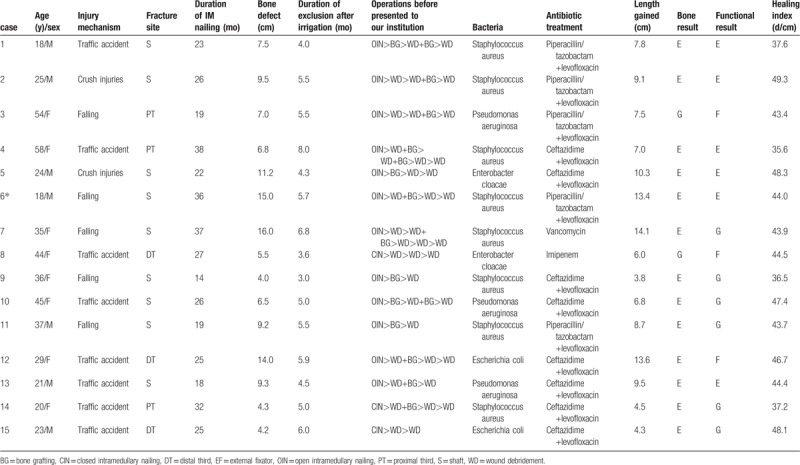
Details and the last results of patients in our present study.

### 2.2 Operative techniques

#### 2.2.1 Step 1: Eradication of infection tissues, restoration of the tissue defects, and continuous irrigation and suction

**Figure 1:**
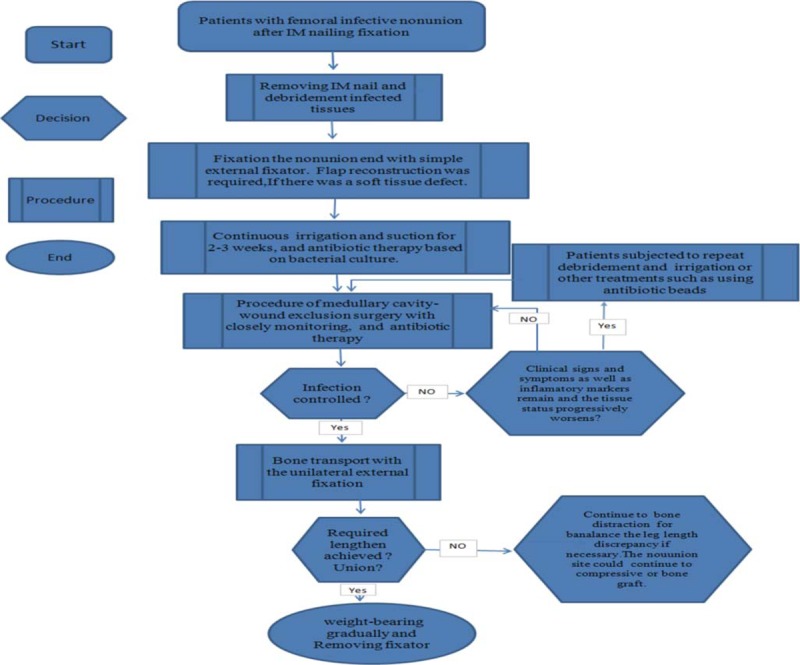
Flow diagram for procedure of surgery.

Preoperative radiographs were taken in the sagittal and coronal planes to assess the bone defect and to plane the application of the unilateral external fixator (Fig. 1). Due to the inconvenience with the existence of the IM nail, the nails were removed first. The procedure of debridement about nonunion area and infected scarred soft tissue was carried out. To fix the nonunion ends, we had applied simple external fixator after removing the IM nail. We cultured the positive fluid, tissues, and infected bone obtained from debridement procedure (Table 1). We re-reamed the medullary canal using a drill after debridement to ensure bone bleeding. Wounds and IM canals were rinsed with pulsed irrigation system and sterilized with iodine complex and normal saline. Then, we placed 2 drainage tubes to ensure proceeding 2 to 3 weeks continuous irrigation with several liters of antibiotic irrigant at the rate of 40 to 60 drops/min (80 mg gentamycin was added per liter of irrigant). All the patients were treated with 2 weeks’ intravenous antibiotics and 4 weeks’ oral antibiotics according to the culture results and sensitivity tests (Table 1). After the infection eradication, bone defect was present in all patients with a mean size of 8.7 cm (rang, 4.0–16.0 cm) as measured on plain radiographs.

#### 2.2.2 Step 2: Procedure of medullary cavity's wound exclusion surgery with closely monitoring and intravenous antibiotics or appropriate oral antibiotics

After the procedure of debridement and 2 to 3 weeks’ continuous irrigation and suction, the patients were followed at 2 weeks, 6 weeks, 3 months, and 6 months and longer. Meanwhile, we were closely monitoring of clinical symptoms or signs, the laboratory results of WBC, CRP, ESR, and X-radiography. If the first debridement and irrigation failed to control the infection, the patient repeated the procedure. No specific criteria were used to determine which patient was to undergo a repeat procedure. Decision-making was individualized, based on persistence or recurrence symptoms or signs of infection or the tissue status progressively worsens, such as persistent wound drainage or nonhealing wound.

#### 2.2.3 Step 3: Bone transport with the unilateral external fixation

Bone transport with the unilateral fixator^[11–13]^ was taken only when the wound had healed, the WBC, ESR, and CRP had returned to the normal levels after the patient did not receive antibiotics for at least 1 month. After a long time of medullary cavity-wound exclusion surgery with closely monitoring, the unilateral consecutive distraction-compression osteosynthesis technique was applied in all patients. Preoperative radiographs were taken in the sagittal and coronal planes to assess the bone situation, to determine the planes of the osteotomies, and to plan the application of the unilateral external fixator. A lateral incision was used in the femoral for osteotomy. Under image intensifier control, 2 or 3 pins (diameter 4.5 mm) were inserted above and below the preselected osteotomy site. To sustain the anatomic axis, each set of pins should be in the same plane and perpendicular to the long axis of the femur. Further pins were inserted if it is necessary. Under direct vision, a series of unilateral drill holes were made in the two-thirds of the circumference of the bones and connected with an osteotome. The unilateral external fixator was attached with a 2 cm gap between it and the leg to allow for swelling. The femoral selected site for osteotomy was exposed superiosteally, and then a transverse osteotomy was made. The periosteum was sutured and the wound was closed with a drainage tube. Thus, the technique of compression-distraction osteogenesis with unilateral external fixator was applied. The latency period was 3 to 5 days after the operation and the rhythm of distraction in corticectomy site to fill defect was 0.25 mm per 9 hours. Continuous passive motion was instituted immediately after the operation to prevent intra-articular adhesions. When the length of bone regeneration had reached about 6.0 cm, the rate of distraction had reduced to 0.25 mm every 12 hours. The rate was adjusted according to the regenerative ability and pain reaction. Physiotherapy started on the second postoperative day, and ROM exercises of the knee and hip were encouraged. Daily shower, including washing the pin sites with antibacterial soap, was encouraged.

During the lengthening, patients were following up every 2 weeks about clinical and radiological. Anteroposterior and lateral radiographs of the femur were taken to monitor bone regeneration, measure the bone loss, and adjust the rate of lengthening. According to the Merianos et al^[14]^ definition of union, when the patient was pain free on full weight bearing and there was radiological evidence of bridging callus across more than 75% of the fracture ends, union was considered to have taken place. And when the required length had been achieved with bony consolidation, the external fixator was removed gradually. Patients could not do weight-bearing during the lengthening. But after the removal of the external fixator, the regenerated bone was protected with patella tendon-bearing braces and crutches, and patients were allowed partial weight-bearing. Full weight-bearing was permitted gradually, considering the amount for hypertrophy of the regenerated bone. We had employed effective and prompt guidance of recovery exercise to 15 patients after surgery, especially after removing the fixator. Function results were ranked as excellent, good, fair, and poor based on the criteria recommend by Paley et al.^[15–18]^

### 2.3 Statistical analysis

All data were analyzed by a commercially available statistical database package (SPSS version 19.0, USA). Enumeration data was described by frequency and measurement data by mean. A paired sample *t* test was used to determine difference in measurement data between preoperative and the last follow-up. The statistical significance level was set at a *P* = .05.

### 2.4 Ethical approval

This study was approved by the Institutional Ethics Committee of the Second Xiangya Hospital of Central South University.

## 3 Results

No patient was lost to following-up in our study. Bone infection was controlled in 12 (80%) patients after initial procedure of debridement and irrigation. Only 3 (20%) patients undergo a repeat procedure. The time of medullary cavity-wound exclusion surgery was ranged from 3 to 8 months (mean 5.2 months). All wounds were healed and no patient required flap coverage. The WBC, ESR, and CRP of our patients were all returned to the normal levels before the application of the unilateral external fixator. Therefore, the method of debridement and irrigation is a compatible choice on the phase of infection-elimination obviously.

**Figure 2:**
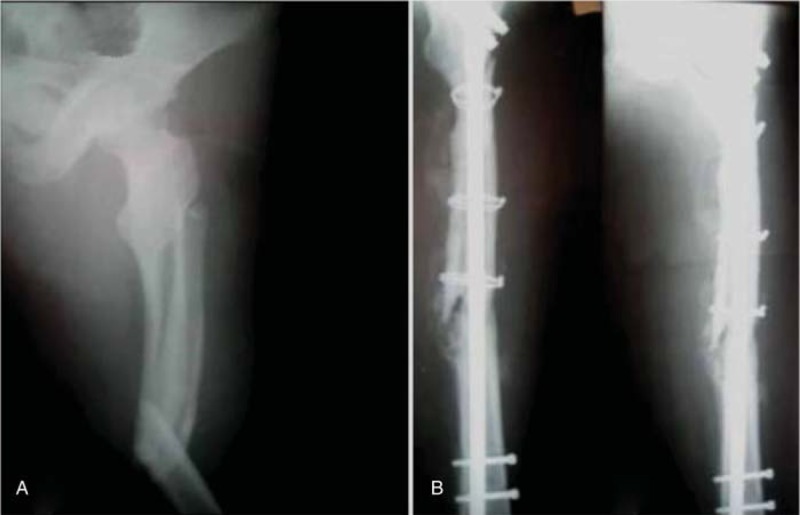
A, Radiograph of a 18-year-old man (case 6) who had an middle femoral shaft fracture. B, Anterioposterior and lateral X-ray of femora after performing the operation of IM nailing merging. IM = intramedullary.

**Figure 3:**
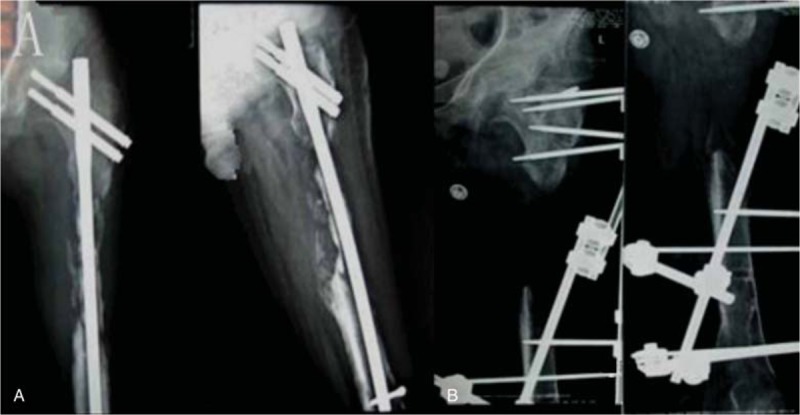
A, The patient was suffering from osteomyelitis and resorption of bone at 36 months later. B, The phase of medullary cavity-wound exclusion surgery after completely debridement and 3 weeks continuous irrigation with several liters of antibiotic irrigant.

**Figure 4:**
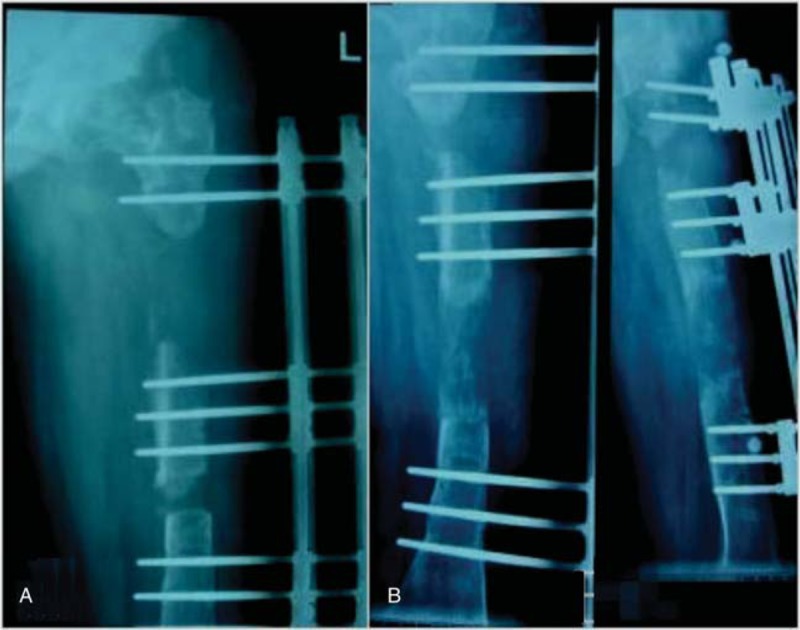
A, After complete debridement and 5.7 months of medullary cavity-wound exclusion surgery, the 3-segment unilateral fixator was applied. B, The callus regenerated well after 10 months of compression-distraction osteogenesis with the external fixator.

**Figure 5:**
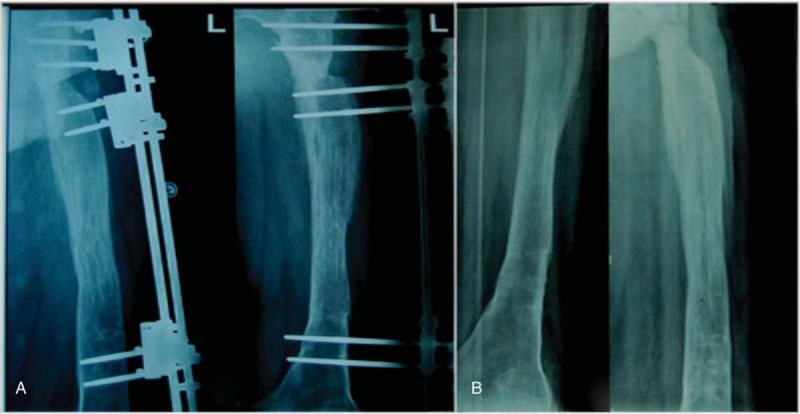
A, The callus regenerated well after 14 months. B, A total lengthening of 15.0 cm was achieved.

Bone union was achieved in all 15 (100%) patients without the recurrence of infection (see Figs. 2–5). The mean external fixation index was 43.4 day/cm (range, 35.6–49.3 days/cm). A residual deformity >7° was present in 4 (26.7%) patients; all patients can walk well without walking aids or braces, 7 (46.7%) cases have the hip and/or knee joint contracture greater than 5°; 9 (60.0%) cases have the ability to perform almost all previous activity of daily living (ADL) with minimal difficulty; 4 (26.7%) patients felt pains (requiring narcotics) after they walked a long distance. According to the evaluation system previously reported by Paley et al,^[16,19]^ bone results were graded as excellent in 13 (86.7%) cases and good in the rest 2 (13.3%) cases, and functional results which are a valuable efficacy variable, were graded as excellent in 6 (40.0%) cases, good in 6 (40.0%), and fair in 3 (20.0%) cases (Table 1). There was no case of femoral head osteonecrosis. The average motion degree of knee in latest follow-up was 94.4°, which was better than the preoperative findings (54.1°) in our 15 cases, the differences had significance (*P* < .05).

Pain was the most common complaint during the distraction period, and it was relieved consistently by oral analgesics. In our study, 8 (53.3%) patients had a pin-track infection, 6 (40%) of our cases had local inflammation, which had been settled with pin care and oral antibiotics, and 2 (13.3%) had a purulent drainage, which had been settled with intravenous antibiotics, During the phase of distraction, loosening of a pin occurred in 4 (26.7%) cases, and all of them were treated by reapplied pins. There were no refractures or neurovascular complications, and no patient had recurrent osteomyelitis.

## 4 Discussion

Even though the procedure of debridement, continuous irrigation, and suction were an accepted treatment modality for treating infection, the rate of success has been variable. Koyonos et al^[20]^ analyzed their clinical results in 136 patients who were treated with irrigation and debridement (I&D) for periprosthetic joint infection with retention of prosthesis, and they suggested that I&D is unlikely to control periprosthetic joint infection. Their data showed that chronic infections should not be treated with I&D, and Staphylococcal organisms were an independent risk for failure of I&D. Sporadically, if an I&D fails to control the infection, one may elect to repeat the procedure. Reviewing the literature, no specific criteria were used to determine which patient should undergo a repeat procedure. Decision-making was individualized. In the study of Byren et al,^[21]^ there are 24 patients who underwent multiple I&D, with the success rate being 25%. While Aboltins et al^[22]^ analyzed their clinical results, there were 12 of 20 patients with staphylococcal periprosthetic joint infection who underwent 2 to 4 I&D and all were treated successfully. In our study, after removing the IM nailing, we placed 2 drainage tubes in patients to ensure proceeding 2 to 3 weeks continuous irrigation and suction with several liters of antibiotic irrigant. Three (20%) patients had a repeated debridement and irrigation, and all wounds were healed and no patient required flap coverage at last. Therefore, we suggested the method of debridement, continuous irrigation, and suction is a compatible choice on the phase of infection-elimination.

Most reports suggested that surgery with IM nailing was an ideal treatment for a patient with long bones fracture. However, Winquist et al^[23]^ suggested that using this method demands the patient be evaluated carefully for associated injuries and be resuscitated adequately. Wolinsky et al^[4]^ showed that as in all methods, problems still arise in IM fixation too. Posttraumatic infected nonunion following IM nailing was one of the most challenging orthopedic problems. Via many documents,^[24,25]^ patients who suffered from infected nonunion of long bones, usually undergo numerous precious surgical interventions, resulting in bone defects, and soft tissue compromise. There were 2 schools of thought in the treatment of infected nonunion, one method was “union-first” and the other was “infection-elimination first”.^[26]^ For “infection-elimination first” treatment, the basic principles included debridement, fracture stabilization, soft tissues reconstruction, and systemic and/or local antibiotic treatment.^[27]^ The surgeons confronted the dilemma of removal or retention of the IM nail in the presence of infection. In the last decade, the literature^[28]^ about the management of infected nonunion has suggested that the 2-stage strategy was the best and well-proven method for infected nonunion. The problem was getting bigger in the presence of IM nail because infection cannot be completely eradicated when the IM nail was in place and infection may spread along the IM canal.^[29]^

Cancellous bone grafting may be an alternative for small bone defects, while larger bone defects require some sort of vascularized grafts. However, refracture and host-graft junction healing problems were common complications with this type of grafting technique. Cierny and Zorn^[30]^ have reported on 44 consecutive patients with segmental debridement defects of the tibia, and made a comparison between the results of treating segmental tibial defects using Ilizarove bone transport and the results of using massive autologous bone graft; they confirmed that the Ilizarove method had lower complication rates. The most disadvantage of our treatment modality is the long-lasting treatment period causing great patients’ discomfort. After operation, it is useful for patients to do systemic functional exercises step by step in the early stage. Bone transport may cause damage to the neurovascular structures particularly in patients with scarring from osteomyelitis and previous failed operations. In our study, achieved bony union without recurrence infection during the follow-up period, and there was no evidence of neurovascular injury in any of our 15 patients. The healing index and overall rate of complications were like those of previous reports in which a circular fixator was used.^[31]^ Based our results (Table 1), we believe that there is no correlation between bone results of healing and the mechanism of injury, the period of bone union as well as external fixator surgery previously.

## 5 Limitation

Our study suffered from some limitations. First, while we presumed surgeons considered their debridement, continuous irrigation and suction thorough, the thoroughness with which each surgeon carried out this procedure cannot be confirmed. However, since our study was carried out by multiple, experienced, fellowship-trained surgeons, our findings may be more generalizable. Second, we reported those patients on oral antibiotics at most recent follow-up only, there was no universal protocol in deciding whether to place patients on oral antibiotics after completing a course of intravenous antibiotics. Third, recall bias may have been introduced. The patients may have misidentified the timing of symptoms in some cases. Fourth, we did not record the preoperative and postoperative range of motion of ankle and hip. The major weakness of our treatment modality is the absence of a control group and our small number of patients.

## 6 Conclusion

Our present study described a safe, effective, and successful alternative technique of bone transport with the unilateral external fixator after debridement, continuous irrigation, and suction for the treatment of the challenging problem of femoral infectious nonunion after IM nailing fixation.

## Acknowledgments

The authors thank all participating patients, as well as the study nurses, co-investigators, and colleagues who made this trial possible.

## Author contributions

XHZ and CFL wrote the draft of the manuscript and participated in the follow-up examination of the patients and clinical material. TL and ZHL performed the surgery, coordinated, and helped to draft and finalize the manuscript. XSZ and YZX participated in the surgical and medical treatment and execution of the study. All authors read and approved the final manuscript.

**Data curation:** Xianghong Zhang, Chunfeng Liu.

**Data analysis and interpretation:** Tang Liu, Xianghong Zhang.

**Funding acquisition:** Tang Liu.

**Methodology:** Xiangsheng Zhang, Zhihong Li, Yaozeng Xu and Tang Liu.

**Project administration:** Tang Liu and Yaozeng Xu.

**Writing – original draft:** Xianghong Zhang and Chunfeng Liu.
